# Waste to Value-Added Product: Developing Electrically Conductive Nanocomposites Using a Non-Recyclable Plastic Waste Containing Vulcanized Rubber

**DOI:** 10.3390/polym13152427

**Published:** 2021-07-23

**Authors:** Amir Hosein Ahmadian Hoseini, Elnaz Erfanian, Milad Kamkar, Uttandaraman Sundararaj, Jian Liu, Mohammad Arjmand

**Affiliations:** 1 School of Engineering, University of British Columbia, Kelowna, BC V1V 1V7, Canada; amir.ahmadian@ubc.ca (A.H.A.H.); milad.kamkar@ubc.ca (M.K.); 2 Department of Chemical and Petroleum Engineering, University of Calgary, Calgary, AB T2N 1N4, Canada; elnaz.erfanian@ucalgary.ca (E.E.); ut@ucalgary.ca (U.S.)

**Keywords:** plastic waste, conductive polymer nanocomposite, carbon nanotube, excluded volume effect, double percolation, electrical conductivity, electromagnetic interference shielding

## Abstract

This study intends to show the potential application of a non-recyclable plastic waste towards the development of electrically conductive nanocomposites. Herein, the conductive nanofiller and binding matrix are carbon nanotubes (CNT) and polystyrene (PS), respectively, and the waste material is a plastic foam consisting of mainly vulcanized nitrile butadiene rubber and polyvinyl chloride (PVC). Two nanocomposite systems, i.e., PS/Waste/CNT and PS/CNT, with different compositions were melt-blended in a mixer and characterized for electrical properties. Higher electrical conduction and improved electromagnetic interference shielding performance in PS/Waste/CNT system indicated better conductive network of CNTs. For instance, at 1.0 wt.% CNT loading, the PS/Waste/CNT nanocomposites with the plastic waste content of 30 and 50 wt.% conducted electricity 3 and 4 orders of magnitude higher than the PS/CNT nanocomposite, respectively. More importantly, incorporation of the plastic waste (50 wt.%) reduced the electrical percolation threshold by 30% in comparison with the PS/CNT nanocomposite. The enhanced network of CNTs in PS/Waste/CNT samples was attributed to double percolation morphology, evidenced by optical images and rheological tests, caused by the excluded volume effect of the plastic waste. Indeed, due to its high content of vulcanized rubber, the plastic waste did not melt during the blending process. As a result, CNTs concentrated in the PS phase, forming a denser interconnected network in PS/Waste/CNT samples.

## 1. Introduction

The current technologies of plastic waste management are not effective nor environmentally friendly [1]. Since 1950, over 5.8 billion tons of plastic waste have been generated globally, which have been accumulated in landfills/natural environment (78.5%), incinerated for energy recovery (13.0%), and recycled (8.5%) [2]. This massive amount of landfill-disposed plastic waste, which is mostly non-biodegradable, has accounted for many ecological and health problems [3,4,5]. The process of waste incineration poses a significant threat to public health and causes environmental damage since it produces lots of toxic air pollutants [6,7]. Recycling plastic waste may address the existing environmental and health concerns. However, frequent recycling of plastics is technically impractical due to deterioration of the final properties of the recycled plastic (e.g., mechanical properties) [8,9]. Moreover, recycling is arduous or even unfeasible for many plastics and polymers, such as contaminated plastics, cured thermoset resins, and vulcanized rubber [10,11]. That is, utilization of plastic waste in different sectors and finding new applications for this material is a promising alternative approach for plastic waste management.

In the case of vulcanized waste rubber such as scrap tires, devulcanization and reclaiming with the aid of thermo-mechanical and chemical processes can be employed to regenerate the rubber into a state that it can be partially mixed, processed, and vulcanized again. Nevertheless, these processes are highly energy intensive, use many chemicals, can cause polymer chain cleavage, and leave behind hazardous waste [12,13]. Instead, pulverization of the rubber into crumb for use as an additive in composite materials is an effective rubber waste treatment method. The major application of the crumb tire rubber is in asphalt paving mixture, concrete, and cementitious materials as a modifier of weight, thermal insulation, and damping property [14,15,16,17,18,19,20]. Crumb rubber is also combined with curatives/binders (e.g., urethane, latex) to manufacture rubber products and thermoplastic rubber blends such as tires, mechanical goods, carpet tiles, mat, artificial turf, and hose at a lower cost [13,21,22,23,24]. However, it sometimes compromises mechanical properties and performance of the final product. For example, Marin-Genesca et al. [25] blended ground tire rubber (GTR) with seven different thermoplastic polymers to be used as industrial work footwear insulation. They reported a dramatic decrease in both mechanical and dielectric properties for GTR loading of 20 wt.% and higher.

Employing plastic waste to develop low-cost conductive polymer composites (CPCs) for electrical-related applications seems to be an appealing idea. The global market share for conductive polymer-based materials is predicted to reach USD 5.7 billion in 2027, primarily fueled by the growing demand for anti-static materials for safe packaging of electronics [26]. To the best of our knowledge, there are just a few studies in the literature on plastic waste-derived CPCs [27,28,29,30,31]. Zribi et al. [30] used recycled polycarbonate and crushed tire rubber to develop carbon black-filled conductive polymer nanocomposites for self-regulated heating and gas sensing applications. They observed a synergy between conductive filler and crushed rubber that resulted in a high-amplitude positive temperature coefficient and an ample sensing response amplitude and selectivity.

Jia et al. [31] converted GTR into a low-cost, flexible electromagnetic interference (EMI) shielding material. They were able to fabricate GTR/CNT composites by mixing GTR particles with carbon nanotube (CNT) at an extremely high speed (2400 rpm) and then molding at a high temperature of 170 °C and a pressure of 50 MPa. They obtained segregated-structured composite with superior electrical conductivity of 109.3 S/m and specific EMI shielding effectiveness of 56 dB/mm at 5.0 wt.% CNT loading. Such a high-performance material could find a huge range of applications in flexible electronic devices; however, segregated morphologies suffer from poor interfacial adhesion [32,33,34], leading to inferior mechanical properties, and their strategy only works for processable plastic waste (i.e., the samples that they were able to mold).

Thus, this study proposes an application for non-processable rubber-based plastic waste by developing value-added conductive polymer nanocomposite at low-cost for a broad range of electrical applications. The waste material is a polymer foam that is used in the fabrication of sporting goods and composed of vulcanized rubber (60 wt.%), polyvinyl chloride (35 wt.%), and additives (5 wt.%). Due to the high content of vulcanized rubber, the plastic waste material did not melt during the mixing process. This property of the waste material induces excluded volume effect which can improve the electrical properties of a CNT-filled polymer nanocomposite through the formation of double percolation morphology. Excluded volume effect happens in a binary system when the filler particles are expelled by one of the phases, and thus concentrate within the other phase. Some examples of non-filler-accommodating phase could be the crystalline phase of a semi-crystalline polymer, non-melting material such as vulcanized rubber, and a polymer with extremely high viscosity or low affinity to the filler. In a system with double percolation morphology, the filler particles form an interconnected conductive network (i.e., percolation) and the minor phase constructs a co-continuous structure. This leads to a reduction in the usage of costly conductive filler and provides more flexibility in the tuning of the final properties of conductive nanocomposite for a specific application of interest [35,36,37,38,39,40,41,42,43,44,45,46,47,48,49,50]. The waste material, carbon nanotube (CNT), and polystyrene (PS) were melt-blended at different compositions, and the compression molded samples were prepared for electrical conductivity, EMI shielding, optical microscopy, and rheological characterizations. The results showed a dramatically lower electrical percolation threshold for CNTs and much higher electrical conduction in the presence of the plastic waste.

## 2. Materials and Methods

### 2.1. Materials and Fabrication

The plastic waste material is a foam with a composition of 35% polyvinyl chloride (PVC), 60% vulcanized nitrile butadiene rubber, and 5% other additives. It was kindly provided by Mustang Survival Corp., an outdoor and sporting goods company, and shredded at the facilities of Native Shoes. The binding polymer is polystyrene (PS) (EA 3130 grade, AmSty LLC, The Woodlands, TX, USA), and the conductive filler is carbon nanotube (NC7000™ Nanocyl S.A., Sambreville, Belgium). The reason for choosing PS as the binding agent is the promising electrical properties obtained for CNT-filled PS nanocomposite in our previous work [51].

**Scheme 1:**
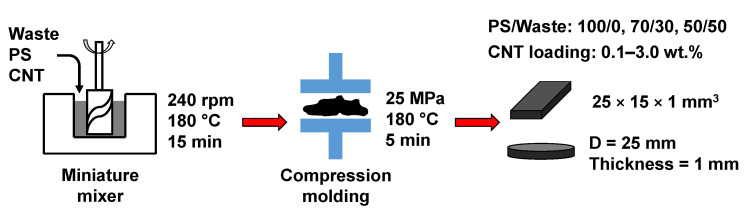
The schematic of sample preparation procedure of the nanocomposites.

Two nanocomposite systems, i.e., PS/CNT and PS/Waste/CNT, were developed. Different blend compositions, including PS/Waste weight ratios of 70/30 and 50/50, and a wide range of CNT loading (0.1–3.0 wt.%) were employed to investigate the effect of using waste material on conductive network formation. The weight percent value of CNT loading is based on the total mass of the polymer matrix, i.e., m_CNT_/(m_PS_ + m_Waste_). The materials were melt-blended altogether simultaneously in an Alberta Polymer Asymmetric Mini-mixer (APAM) [52] for 15 min at 240 rpm and 180 °C. Performing the melt mixing process at higher temperature was not feasible since the vulcanized rubber in the waste material starts to burn (see thermogravimetric analysis of the materials in Figure S1 of the Supplementary Materials). The melt-blended samples were collected and solidified in the air and then compression molded into rectangular slabs (25 × 15 × 1 mm^3^) and circular discs (diameter: 25 mm, thickness: 1 mm) using a hydraulic hot press (4389, Carver Inc., Wabash, IN, USA). The compression molding process was carried out at 180 °C under 25 MPa pressure for 5 min followed by cooling with water. The sample preparation procedure is shown schematically in Scheme 1.

### 2.2. Characterization

The impact of the waste material on the thermal stability of the nanocomposites was evaluated by conducting thermogravimetric analysis (TGA) on PS and PS/Waste samples using TGA Q500 instrument (TA Instruments, New Castle, DE, USA) at a heating rate of 20 °C/min from room temperature to 900 °C under nitrogen atmosphere. The TGA results are presented in Figure S1 of the Supplementary Materials. Fourier transform infrared (FTIR) spectrum of the waste material was recorded on a Nicolet™ iS20 Fourier Spectrometer (ThermoFisher SCIENTIFIC, Waltham, MA, USA)in a range of 400–4000 cm^−1^ and the results are presented in Figure S2 of the Supplementary Materials. The particle size distribution of the plastic waste material was provided in Figure S3 of the Supplementary Materials.

Optical microscopy was employed to evaluate the dispersion state of CNTs within the developed nanocomposites qualitatively for the samples with 1.0 wt.% CNT loading. First, the compression molded samples were cut into thin sections of 5-μm thickness using Leica ultramicrotome EM UC6 (Leica Biosystems, Nussloch, Germany). Olympus BX60 optical microscope (Olympus Inc., Center Valley, PA, USA) featured with Olympus DP80 camera was used in transmission mode to capture images of the prepared sections at different magnifications.

The rheological behavior of the developed nanocomposites was evaluated using a rheometer (MCR 102, Anton Paar, Graz, Austria) equipped with a 25 mm parallel-plate geometry. The gap size was fixed at 0.8 mm, and the temperature was set at 180 °C. The oscillatory frequency sweep tests were performed at a strain amplitude of 1% on PS and PS/Waste samples with 1.0 wt.% CNT loading and without CNT.

The compression molded samples were characterized for electrical properties. DC electrical conductivity measurement was carried out using Loresta-GX (MCP-T700 with 4-pin ESP probe, Nittoseiko Analytech Co., Yamato, Tokyo, Japan) and Hiresta-GX (MCP-HT800 with circular URS probe, Nittoseiko Analytech Co., Yamato, Japan) resistivity meters for samples with high and low conductivity, respectively. Electromagnetic interference (EMI) shielding performance of the nanocomposites was investigated over the X-band frequency range (8.2–12.4 GHz) using a vector network analyzer (P937xA, Keysight Technologies, Santa Rosa, CA, USA). The outputs of a two-port vector network analyzer setup are scattering parameters (S-parameters), which will be used to obtain EMI shielding properties as follows:

R = P_R_/P_I_ = |S_11_|^2^ = |S_22_|^2^, (1)

T = P_T_/P_I_ = |S_21_|^2^ = |S_12_|^2^, (2)

A = P_A_/P_I_ = 1 − (R + T), (3)

EMI SE_R_ = 10 log(1/(1 − R)), (4)

EMI SE_A_ = 10 log ((1 − R)/T), (5)

EMI SE_T_ = EMI SE_R_ + EMI SE_A_ = 10 log (P_I_/P_T_), (6)

where S_11_ and S_22_ are the reflected voltage magnitude divided by the incident voltage magnitude in ports 1 and 2, respectively; S_12_ is the transmitted voltage magnitude from port 2 to port 1 divided by incident voltage magnitude in port 2 and S_21_ is the transmitted voltage magnitude from port 1 to port 2 divided by incident voltage magnitude in port 1. P_I_, P_R_, P_A_, and P_T_ are the power of incident, reflected, absorbed, and transmitted wave; R, A, and T are called reflectance, absorbance, and transmittance coefficients; EMI SE_R_ and EMI SE_A_ are EMI shielding effectiveness of reflection and absorption. The total EMI shielding effectiveness (EMI SE_T_) of the sample is obtained by the summation of EMI SE_R_ and EMI SE_A_. EMI SE values are expressed in dB and show the effectiveness of the sample in shielding of the EM waves, while R and A values define the dominant EMI shielding mechanism. To study the EMI shielding performance of a sample, it is recommended to investigate these parameters simultaneously [53].

## 3. Results and Discussion

### 3.1. Optical Microscopy

**Figure 1:**
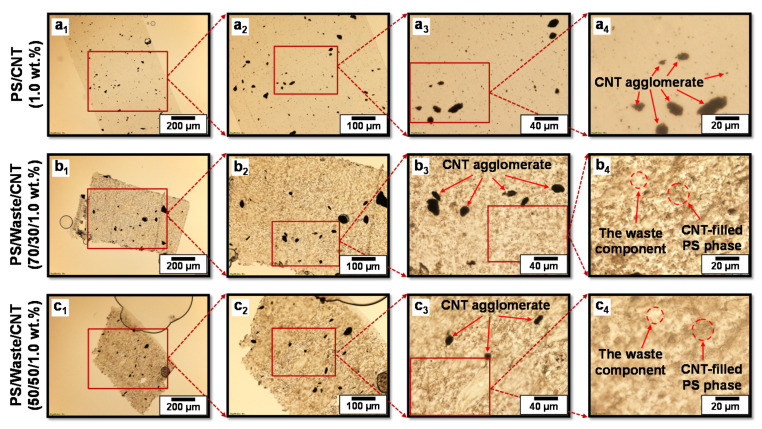
Optical microscopy images of (**a_1_**–**a_4_**) PS/CNT (1.0 wt.%), (**b_1_**–**b_4_**) PS/Waste/CNT (70/30/1.0 wt.%), and (**c_1_**–**c_4_**) PS/Waste/CNT (50/50/1.0 wt.%) nanocomposites at different magnification.

Figure 1 shows optical microscopy images taken from thin sections (5 μm thickness) of PS/CNT and PS/Waste/CNT nanocomposites with 1.0 wt.% CNT loading. The PS/CNT sample contains copious CNT agglomerates of different sizes ranging from a few μm up to 30 μm. Incorporation of the waste material in the blend formulation improved the dispersion state of CNTs, so that by increasing the waste material content to 50 wt.%, both the number and size of agglomerates decreased. Based on percolation theory, systems with better dispersion of the conductive filler are expected to form the conductive network more readily. However, many studies have revealed that some level of agglomeration is vital to effective conductive network formation in filled polymer nanocomposites [51,54,55,56,57].

In the PS/Waste/CNT samples, a co-continuous morphology of light and dark phases is observed; the latter one is the PS phase hosting CNT particles. The waste material did not melt during the melt mixing process. Therefore, no CNT particle could penetrate this phase, so it appears lighter on optical images compared to the CNT-filled PS phase. By zooming in on the optical images of PS/Waste/CNT samples, many tiny CNT agglomerates are observed within the darker phase, which proves that the darker phase is PS filled with CNTs. These tiny CNT agglomerates are also evident in the optical images of PS/CNT sample. The co-continuous morphology with CNTs in one of the phases may lead to a double percolation structure, which significantly enhances conductive network formation and electrical conduction in PS/Waste/CNT nanocomposites.

### 3.2. Rheology

It is well known that dynamic moduli in the low-frequency region are very responsive to any changes in the microstructural features of filled polymer nanocomposites. Recently, it was shown that among dynamic moduli, storage modulus (G′) is more sensitive to subtle changes in the internal structure of nanocomposites compared to loss modulus G′′ [58]. The change in rheological response of polymeric systems upon introduction of a filler stems from filler–polymer and filler–filler interactions [59,60]. These interactions affect both local motion (Rouse regime) and long-range arrangement (chain reptation) of polymer chains, leading to changes in rheological response. Based on the literature [61], the impact of the existence of a secondary component in a polymeric matrix on long-range motions is more pronounced, leading to a change in the slope of dynamic moduli at low frequencies. Hence, masking the long-range motions upon the incorporation of a filler can be detected by any deviation from terminal behavior (i.e., G′ ∝ ω^2^ and G′′ ∝ ω^1^). That is, the extent of network formation of a secondary component in a polymer can be qualitatively evaluated by the slope of the dynamic moduli in the low-frequency region. In this regard, the stronger the network structure, the lower the slope of the dynamic moduli, signifying a better dispersion quality of the filler and/or better filler–polymer interactions.

**Figure 2:**
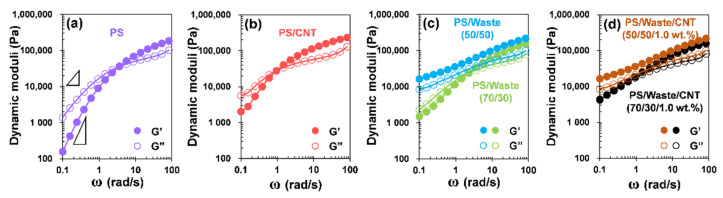
Storage G’ (solid circles) and loss G” (open symbols) moduli of (**a**) PS, (**b**) PS/CNT, (**c**) PS/Waste and (**d**) PS/Waste/CNT as a function of angular frequency at a fixed strain amplitude of $\gamma_{0}$ = 1% at 180 °C.

Herein, the effect of the addition of the waste material on rheological properties of pure PS and PS/CNT (1.0 wt.%) nanocomposite was investigated. Figure 2 shows the dynamic moduli of the samples as a function of frequency at a locked strain amplitude of $\gamma_{0}=1\%$. The neat PS sample showed a terminal behavior (see Figure 2a), signaling the relaxation of polymer chains in the low-frequency region. As expected, dynamic moduli increase upon the introduction of CNTs. As can be seen in Figure 2b, the increase in the storage modulus is sharper than the rise in loss modulus. This confirms the higher sensitivity of the storage modulus to any changes in the microstructure of the samples, which is in line with our previous work [58]. Although the extent of increase in storage modulus is greater than loss modulus, the storage modulus is still smaller than loss modulus (i.e., G′ < G″) in the presence of 1.0 wt.% CNTs, indicative of a liquid dominant response. This confirms the inability of CNTs at the mentioned concentration to form an interconnected, mechanically robust network structure throughout the PS matrix. This is in good harmony with the electrical conductivity results; see next section, in which PS/CNT (1.0 wt.%) is not in the electrically conductive zone.

Interestingly, incorporation of 30 wt.% waste material significantly enhances the rheological properties, and the storage modulus increased one order of magnitude compared to values for PS/CNT 1.0 wt.% (compare Figure 2a–c). Increasing the content of the waste material from 30 to 50 wt.% led to further increment in the values of the dynamic moduli and emergence of “pseudo-solid” like properties, i.e., G’ > G”. The higher elastic contribution in the presence of the waste material, which does not melt at the experiment temperature, verifies that this component forms a 3D network structure throughout the bulk of the PS matrix.

Introduction of CNTs to PS/Waste (70/30) improves the storage modulus from 1.4 to 4.3 kPa, signaling synergistic effect of waste component and CNT on rheological parameters via double network formation. However, the network structure of the waste component at the high concentration (i.e., 50 wt.%) is strong enough that the effect of CNT incorporation on rheological properties is negligible (compare Figure 2c,d). In other words, the secondary network of CNTs established in the PS phase is masked by the structure formed by 50 wt.% waste components. Thus, CNTs’ network does not add any mechanical properties to PS/Waste (50/50) systems. However, as a direct result of excluded volume effect due to the existence of non-melting waste component (i.e., 50 wt.%), dispersed CNTs in the PS phase formed a much denser network structure consisting of a higher number of tube-tube direct contact, leading to a dramatic increase in electrical conductivity (see next section). To recapitulate, the waste material not only contributes significantly to electrical conductivity enhancement of PS/Waste/CNT systems through exclude volume effect but also significantly increased the rheological properties.

### 3.3. DC Electrical Conductivity

**Figure 3:**
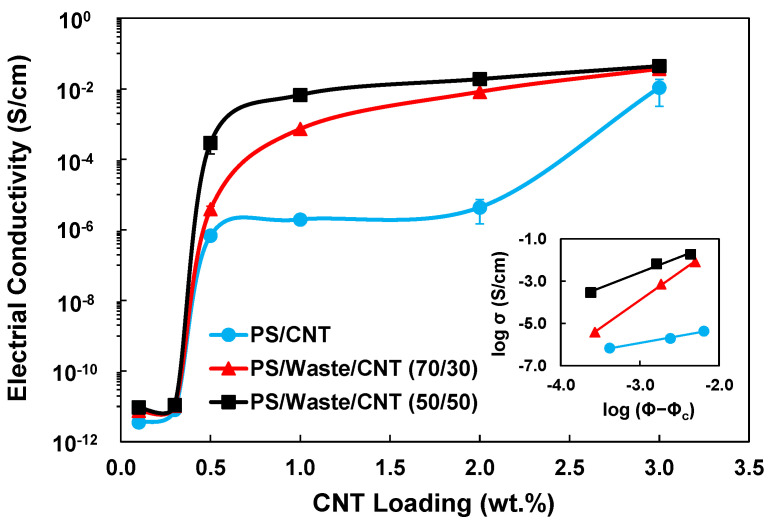
Electrical conductivity of PS/CNT and PS/Waste/CNT nanocomposites at CNT loadings of 0.1–3.0 wt.%, and PS/Waste weight ratio of 70/30 and 50/50. The inset shows the linear fitting of the percolation model (Equation (7)) to the electrical conductivity data. σ is electrical conductivity; Φ is filler volume fraction; Φ_c_ is percolation threshold.

Electrical conductivity of an insulative polymer matrix filled with a well-dispersed conductive filler dramatically depends on filler content following a sigmoid trend, widely known as the percolation curve. Figure 3 shows the DC electrical conductivity of the compression molded PS/Waste/CNT and PS/CNT samples at different loadings of CNT and two ratios of PS:Waste. The electrical conductivity of the pristine materials is 3.98 × 10^−12^ S/cm for the plastic waste, 4.13 × 10^−16^ S/cm for PS, and 10^4^ S/cm for CNT. A typical S-shape trend (i.e., percolation behavior) is observed for all nanocomposites. At low CNT loadings (e.g., 0.1 and 0.3 wt.%) the samples are insulative with electrical conductivities of less than 1 × 10^−11^ S/cm. By increasing the CNT loading to 0.5 wt.%, electrical conductivity leaps up several orders of magnitude beyond the percolation threshold and then reaches a plateau at higher CNT loadings. The percolation threshold is defined as the specific filler loading at which the first conductive path is formed inside the polymer matrix through which electrons can flow readily. The characteristics of the percolation curve, such as percolation threshold, the extremeness of the sudden jump, and the level of plateau region, determine the quality of the network formed by the conductive filler particles.

According to the percolation theory, a power-law equation correlates electrical conductivity of filled polymer nanocomposites with filler content beyond the percolation threshold, as follows [62]:

σ = σ_0_ (Φ − Φ_c_)^t^    for Φ > Φ_c_, (7)

where σ is electrical conductivity; Φ is filler volume fraction; Φ_c_ is percolation threshold; σ_0_ is a scaling factor related to the conductivity of filler, contact resistance of filler particles, and network topology; t is called the critical exponent and depends on the connectivity of the system [63,64]. The theoretical value of critical exponent t varies between 1.33 and 2, while the experimental values for CNT-filled polymer nanocomposites reported in the literature range from 0.7 to 3.1 [65,66,67,68,69,70]. Although not well understood, this discrepancy was attributed to the tunneling conduction mechanism, inherent conductivity of CNTs, complex morphology of CNT bundles, and structural flexibility of CNTs [71]. Percolation model’s parameters are obtained by linear regression of Log(σ) against Log(Φ−Φ_c_) that maximizes correlation coefficient (R-squared) with respect to Φ_c_. Table 1 presents the percolation parameters fitted to the electrical conductivity data of the developed nanocomposites.

Electrical conductivity measurement revealed better network formation of CNTs in PS/Waste/CNT samples compared to its other counterparts without waste component. The calculated percolation threshold for PS/Waste/CNT (50/50) nanocomposite is 0.115 vol.%, almost 10% and 30% lower than that of PS/Waste/CNT (70/30) and PS/CNT nanocomposites, respectively (see Table 1). This implies that in the presence of the waste material, CNT particles percolate more readily so that the initiation of conductive network happens at a lower CNT loading. Additionally, the plateau of the percolation curve for PS/Waste/CNT samples reaches above 1 × 10^−2^ S/cm, while for PS/CNT samples, it is slightly higher than 1 × 10^−6^ S/cm. More importantly, as shown in Figure 3, nanocomposite with higher waste content shows a sharper percolation behavior with a higher plateau. That is, at all CNT loadings, the electrical conductivity of the nanocomposites increases with increasing the ratio of the waste component, i.e., σ_PS/Waste/CNT (50/50)_ > σ_PS/Waste/CNT (70/30)_ > σ_PS/CNT_.

Interestingly, the electrical conductivity of PS/CNT nanocomposites exhibits a secondary jump after plateau, approaching the electrical conductivity of PS/Waste/CNT (3.0 wt.%) nanocomposites. This could be attributed to poor dispersion and a higher number of isolated agglomerates of CNTs in PS/CNT samples, corroborated by imaging techniques (see Figure 1), which hinders the formation of an interconnected network throughout the polymeric media at low CNT loadings.

Enhanced network of CNTs in PS/waste/CNT nanocomposites compared to PS/CNT is attributed to double percolation morphology, which resulted from the excluded volume effect of the waste material. Due to the high content of vulcanized rubber (60%), the waste material is not meltable, and it stayed solid during melt blending of the nanocomposites. Accordingly, the volume occupied by the waste material could not accommodate any CNTs (i.e., excluded volume), and CNTs get expelled and concentrated into the continuous PS phase. In other words, in the presence of the waste material, CNTs have access to less volume to get dispersed in, which increases the chance of tube-tube contacts, promoting better network formation and, thus, higher electrical conduction at a given CNT loading. For instance, the PS/Waste (50/50) filled with 0.5 wt.% CNT conducts electricity 150 times higher than PS filled with 1.0 wt.% CNT. It is worth mentioning that CNT concentration in the PS phase of these two nanocomposites is the same, considering that CNTs get dispersed only in the PS phase.

Double percolation is achieved when selective localization and confinement of the conductive filler within a co-continuous structure (e.g., phase or interface of binary blends) leads to significant enhancement in the conductive network. Many studies tried to develop such an excluded volume-based morphology in immiscible polymer blends [35,36,37,38,39,40,41,42,43,44,45,46,47,48,49,50] and semi-crystalline polymers [72,73,74,75] through modification of processing condition, blend composition, and thermodynamic affinity between polymers and filler. The advantage of our strategy over other methods is achieving a percolated structure of CNTs by a simple melt mixing process with no need of any modification or introduction of any other expensive polymeric component.

### 3.4. Electromagnetic Interference (EMI) Shielding

EMI shielding is a property of interest for applications in which the protection of electronic devices against damaging electromagnetic (EM) waves is needed. Performance of a filled polymer nanocomposite in shielding of EM waves depends on the electrical and magnetic properties of the components, microstructure and morphology of the nanocomposite, and, more importantly, level of conductive network formation [76,77,78]. Two main mechanisms by which a material attenuates an incident EM wave’s power are reflection and absorption. Reflection happens when there is a high impedance mismatch between two media. The intrinsic impedance of a material is defined by $\sqrt{\frac{j\omega \mu }{\sigma +j\omega \varepsilon }}$ where σ is electrical conductivity, ε is dielectric permittivity, μ is magnetic permeability, ω is angular frequency of the EM wave, and j is the imaginary unit. The greater number of surface nomadic charges leads to higher impedance mismatch between the shield and air (as the default medium), promoting reflection of the incident EM wave. For this reason, metals are widely used as an effective shield to reflect EM waves due to their high electrical conductivity. Absorption in filled polymer nanocomposites originates mainly from ohmic loss and polarization loss. The latter one contributes to a minor extent, and it becomes significant when a filler with a high dielectric constant like ceramic is used. Ohmic loss is the dissipation of energy caused by the movement of free charges through the conductive paths.

**Figure 4:**
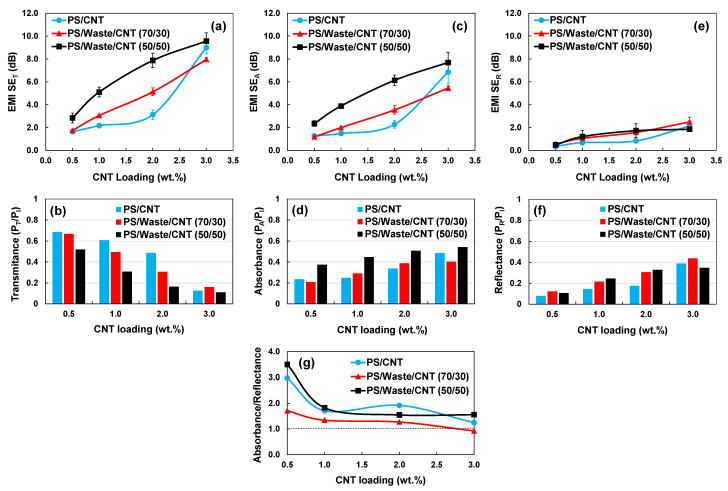
EMI shielding performance of PS/CNT and PS/Waste/CNT nanocomposites at different CNT loadings and the waste material content, (**a**) Total EMI SE, (**b**) Transmittance, (**c**) EMI SE of absorption, (**d**) Absorbance, (**e**) EMI SE of reflection, (**f**) reflectance, (**g**) Absorbance/Reflectance ratio.

Figure 4 shows EMI shielding effectiveness (EMI SE), shielding reflectance (R), shielding absorbance (A), and A/R plots for the developed nanocomposites. Due to an extremely low level of electrical conduction (less than 1 × 10^−11^ S/cm), samples containing 0.1 and 0.3 wt.% CNT did not exhibit any shielding properties; therefore, their results are not shown here for brevity. Electrical conductivity is an important factor in the EMI shielding performance of polymer nanocomposites. For example, electrical conductivity and EMI SE_T_ of PS/Waste/CNT (50/50) with 0.5 wt.% CNT content was measured 2.95 × 10^−4^ S/cm and 2.84 dB, which increased to 4.40 × 10^−2^ S/cm and 9.55 dB at 3.0 wt.% CNT loading (see Figure 3 and Figure 4a). In other words, PS/Waste/CNT (50/50/0.5) shielded less than 50% of the incident EM wave’s power, while this value for higher conductive PS/Waste/CNT (50/50/3.0) is almost 90% (see Figure 4b).

By increasing CNT loading, a better conductive network is formed (see Figure 3) and the number of free charges increases. Accordingly, in the samples with higher CNT content, more electrons find longer conductive paths to flow through in each half cycle of the alternating field, leading to higher dissipation of electrical energy (i.e., higher absorption loss) [78,79,80]. Figure 4c,d (EMI SE_A_ and absorbance) clearly show this trend for all the nanocomposites. The addition of a conductive filler such as CNT to an insulative polymer increases the impedance mismatch of the polymer nanocomposite with respect to air and, thus, higher EMI SE_R_ is expected upon increasing CNT loadings. Nevertheless, the limiting factor for EMI shielding reflection enhancement in a filled polymer nanocomposite is the intrinsic insulating nature of polymers (as the major phase), which is associated with low number of interacting surface nomadic charges to reflect EM wave. This explains why EMI SE_R_ of the nanocomposites (Figure 4e) did not change much with CNT loading (almost 2.5 dB increase over 0.5–3 wt.% range of CNT loading), though reflectance increased from 0.1 to around 0.4 (Figure 4f). Based on absorbance and reflectance values (see Figure 4d,f), the dominant mechanism of EMI shielding in the developed nanocomposites is absorption, and by increasing CNT loading, the contribution ratio of reflection to absorption increases (see Figure 4g).

**Scheme 2:**
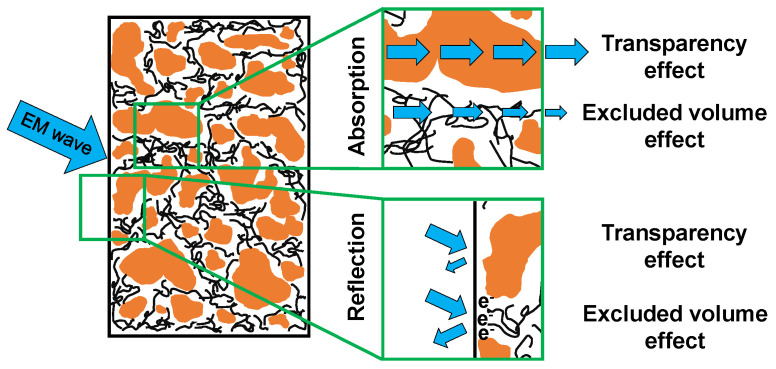
The schematic of excluded volume and transparency effects of the waste material on EMI shielding performance (absorption and reflection) of PS/Waste/CNT nanocomposite. The white and orange phases represent PS and the waste material, respectively.

It was observed that excluded volume effect of the waste material enhanced the network formation and electrical conduction in PS/Waste/CNT nanocomposites. This effect promotes both absorption and reflection shielding by increasing ohmic loss and impedance mismatch of the CNT-containing PS phase, respectively. Thus, higher EMI shielding by absorption and reflection mechanisms is expected at higher content of the waste material, which is not the case for PS/CNT and PS/Waste/CNT (70/30) samples at 0.5 and 3.0 wt.% CNT loadings (Figure 4c–f). In fact, the waste material phase is transparent to the EM wave since it is an insulator and does not accommodate any CNT particles. This adversely affects the EMI shielding reflection by reducing the overall impedance mismatch of the nanocomposite. Moreover, in a sample with higher content of the waste material, there is a higher chance that the penetrating EM wave seeps through the transparent phase of the waste material and leaves the sample without losing much power, which results in lower EMI shielding absorption. These two effects of the waste material (i.e., excluded volume and transparency) on the shielding of the incident EM wave are shown schematically in Scheme 2.

In this regard, there is a trade-off between excluded volume and transparency effects of the waste material on the EMI shielding performance of the PS/Waste/CNT nanocomposites. For example, at a low CNT loading of 0.5 wt.%, there is a lack of an effective conductive network in both PS/CNT and PS/Waste/CNT (70/30) nanocomposites (see Figure 3). So, the detrimental transparency effect of the waste material overshadowed the excluded volume effect on EMI shielding absorption in PS/Waste/CNT (70/30/0.5) sample. This resulted in lower EMI SE_A_ and absorbance compared to PS/CNT (0.5 wt.%) (see Figure 4c,d). However, further increase in the waste material content led to the formation of a well-established conductive network in the PS/Waste/CNT (50/50/0.5) sample (see Figure 3), and the excluded volume effect of the waste material became dominant. The excluded volume effect of the waste material was significant enough so that PS/Waste/CNT (50/50/0.5) shows the highest EMI SE_A_ and absorbance values among PS/CNT (0.5 wt.%) and PS/Waste/CNT (70/30/0.5) nanocomposites.

Another discrepancy observed in EMI shielding performance of the nanocomposites at 3.0 wt.% CNT loading can be justified analogously. Hence, although the electrical conductivity of the samples increases via excluded volume effect by increasing the loading of the waste component in PS/Waste/CNT nanocomposites at all concentrations of the nanotubes, the EMI SE of the samples did not show the same dependency on concentration of the waste, attributed to its transparency effect.

## 4. Conclusions

Electrically conductive carbon nanotube (CNT)-filled polymer nanocomposites were developed using a non-recyclable plastic waste material that is currently being sent to landfill. Due to the high content of vulcanized rubber (60 wt.%), the plastic waste material is not meltable, and thus melt blending of CNT and this waste material (as the host matrix) was not feasible. Our proposed strategy is melt mixing a host thermoplastic polymer (polystyrene in this study) with the waste material as a secondary filler along with CNT as the conductive filler. This approach took advantage of non-melting property of the waste material to introduce excluded volume effect, leading to the formation of a denser network of CNTs via double percolation structuring. It was observed that PS/Waste/CNT nanocomposites with double percolation morphology, which was evidenced by rheological and image analysis results, featured significantly better percolation of CNT particles and higher electrical conduction compared to the PS/CNT nanocomposite. Moreover, incorporation of the plastic waste material greatly improved the performance of the developed nanocomposites in shielding electromagnetic waves.

The significance of using the plastic waste material is not only turning a waste into a value-added product but also decreasing the amount of polymeric matrix and CNT needed to obtain a specific level of electrical conductivity, which has a huge impact on the final cost of the nanocomposite. Moreover, utilization of the plastic waste provides more flexibility in tuning electrical conductivity of the nanocomposite for a variety of applications requiring different levels of electrical conductivity just by small changes in the blend composition, i.e., PS/Waste ratio and CNT loading. To recapitulate, this study demonstrated the potential of using a non-recyclable vulcanized plastic waste material in the development of value-added conductive polymer nanocomposite at a lower cost.

## Figures and Tables

**Table 1:** Fitted percolation parameters of the power-law model (Equation (7)).

Nanocomposite	Φ_c_ (vol.%)	*t*	σ_0_ (S/cm)	R-Squared
PS/CNT	0.160	0.665	1.838 × 10^−4^	0.993
PS/Waste/CNT (70/30)	0.132	2.638	1.049 × 10^4^	0.999
PS/Waste/CNT (50/50)	0.115	1.458	6.150 × 10^1^	0.990

## Data Availability

The dataset generated and/or analyzed during the current study are available from the corresponding author on reasonable request.

## Supplementary Materials

The following are available online at https://www.mdpi.com/article/10.3390/polym13152427/s1, Figure S1: Thermogravimetric analysis (TGA) of PS and PS/Waste composites under nitrogen gas flow and temperature ramp of 20 °C/min from room temperature to 900 °C, (a) weight loss percentage, and (b) derivative of weight loss over temperature, Figure S2: Fourier transform infrared (FTIR) spectrum of the plastic waste material, Figure S3: Particle size distribution of the plastic waste material.
