# Supplementary material for: Waste to Value-Added Product: Developing Electrically Conductive Nanocomposites Using a Non-Recyclable Plastic Waste Containing Vulcanized Rubber
Source: Polymers (Basel). 2021 Jul 23;13(15):2427. doi: 10.3390/polym13152427 (PMC8348210; doi:10.3390/polym13152427)
Supplement: Supplementary file 1 [file polymers-13-02427-s001.zip › polymers-1306539-supplementary.pdf]

Supplementary Materials

Waste to value-added product: Developing electrically conductive nanocomposites using a non-recyclable plastic waste containing vulcanized rubber

Amir Hosein Ahmadian Hoseini [1], Elnaz Erfanian [2], Milad Kamkar [1], Uttandaraman Sundararaj [2], Jian Liu [1,]* and Mohammad Arjmand [1,]*

[1] School of Engineering, University of British Columbia, Kelowna, British Columbia, V1V 1V7, Canada; amir.ahmadian@ubc.ca (A.A.); milad.kamkar@ubc.ca (M.K.)
[2] Department of Chemical and Petroleum Engineering, University of Calgary, Calgary, Alberta, T2N 1N4, Canada; elnaz.erfanian@ucalgary.ca (E.E.); ut@ucalgary.ca (U.T.)
* Correspondence: jian.liu@ubc.ca (J.L.); mohammad.arjmand@ubc.ca (M.A.)

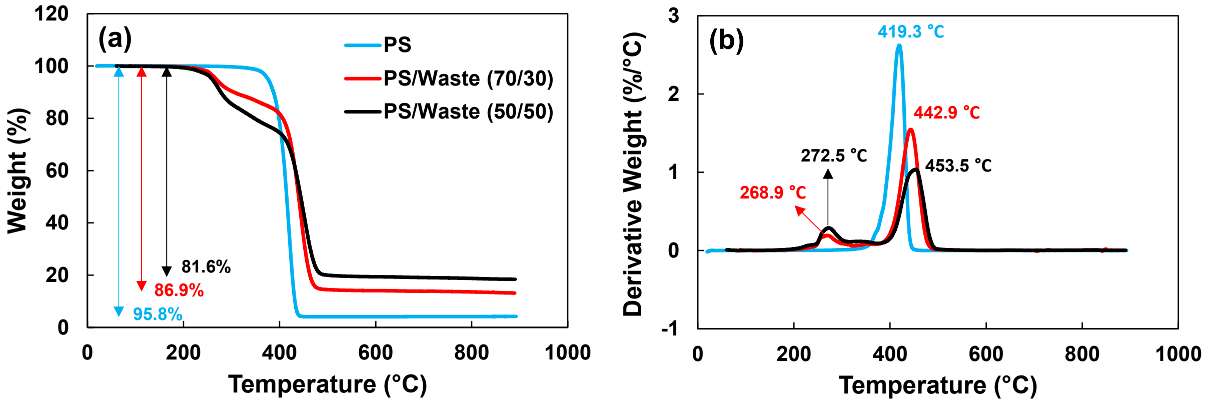

Figure S1: Thermogravimetric analysis (TGA) of PS and PS/Waste composites under nitrogen gas flow and temperature ramp of 20 °C/min from room temperature to 900 °C, (a) weight loss percentage, and (b) derivative of weight loss over temperature.

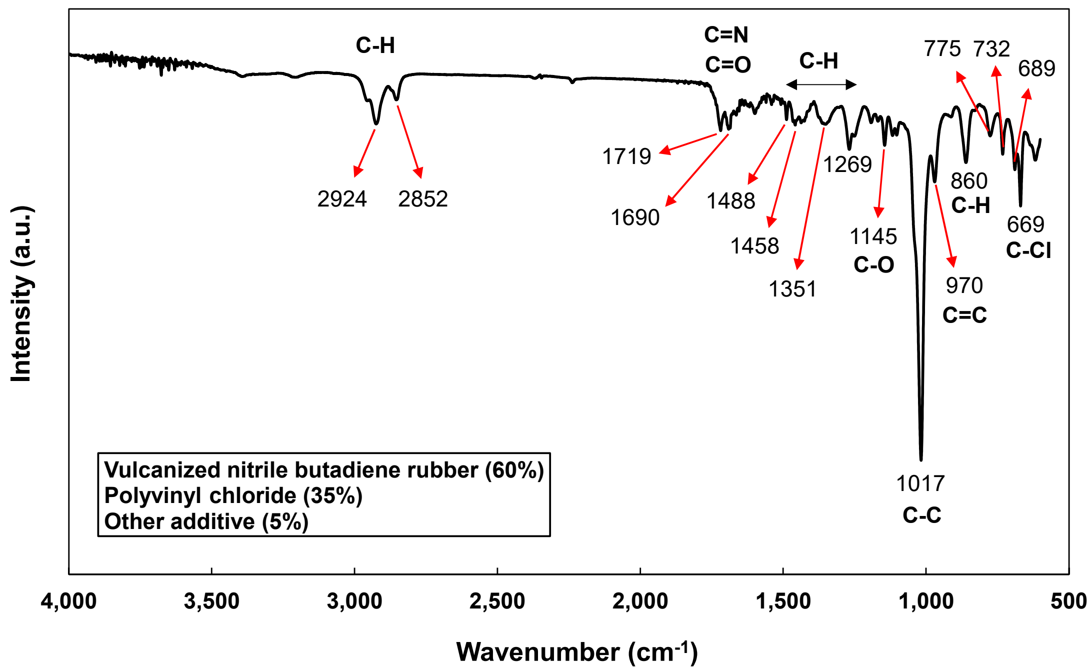

Figure S2: Fourier transform infrared (FTIR) spectrum of the plastic waste material.

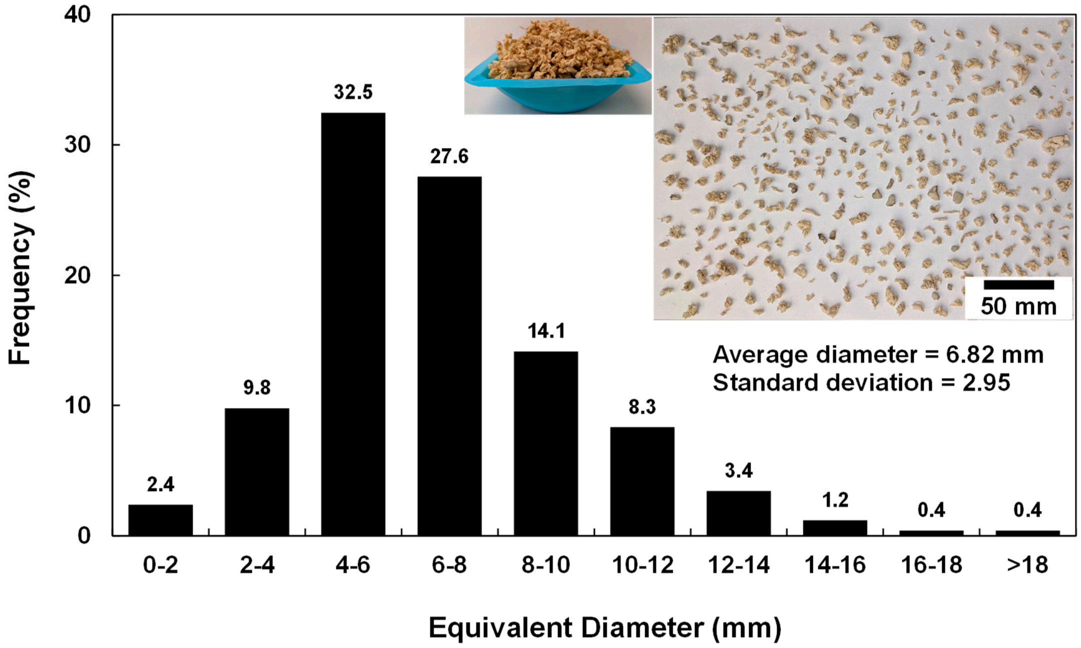

Figure S3: Particle size distribution of the plastic waste material. ImageJ software package is used to perform image analysis.
